# High‐density genetic variation maps reveal the correlation between asymmetric interspecific introgressions and improvement of agronomic traits in Upland and Pima cotton varieties developed in Xinjiang, China

**DOI:** 10.1111/tpj.14760

**Published:** 2020-05-04

**Authors:** Xinhui Nie, Tianwang Wen, Panxia Shao, Binghui Tang, Aini Nuriman‐guli, Yu Yu, Xiongming Du, Chunyuan You, Zhongxu Lin

**Affiliations:** ^1^ Key Laboratory of Oasis Ecology Agricultural of Xinjiang Bingtuan Agricultural College Shihezi University Shihezi Xinjiang 832000 China; ^2^ National Key Laboratory of Crop Genetic Improvement College of Plant Science and Technology Huazhong Agricultural University Wuhan Hubei 430070 China; ^3^ Cotton Research Institute Shihezi Academy of Agriculture Science Shihezi Xinjiang 832000 China; ^4^ Cotton Research Institute Xinjiang Academy of Agriculture and Reclamation Science Shihezi Xinjiang 832000 China; ^5^ State Key Laboratory of Cotton Biology Institute of Cotton Research Chinese Academy of Agriculture Science Anyang Henan 45500 China

**Keywords:** *Gossypium hirsutum*, *Gossypium barbadense*, genetic introgression, selection, fibre traits, GWAS

## Abstract

The two new world tetraploid cottons, *Gossypium hirsutum* and *Gossypium barbadense*, are cultivated worldwide and are characterised by a high yield and superior fibre quality, respectively. Historical genetic introgression has been reported between them; however, the existence of introgression and its genetic effects on agronomic traits remain unclear with regard to independent breeding of *G. hirsutum* (Upland cotton) and *G. barbadense* (Pima cotton) elite cultivars. We collected 159 *G. hirsutum* and 70 *G. barbadense* cultivars developed in Xinjiang, China, along with 30 semi‐wild accessions of *G. hirsutum*, to perform interspecific introgression tests, intraspecific selection analyses and genome‐wide association studies (GWAS) with fibre quality and yield component traits in multiple environments. In total, we identified seven interspecific introgression events and 52 selective sweep loci in *G. hirsutum*, as well as 17 interspecific introgression events and 19 selective sweep loci in *G. barbadense*. Correlation tests between agronomic traits and introgressions showed that introgression loci were mutually beneficial for the improvement of fibre quality and yield traits in both species. In addition, the phenotypic effects of four interspecific introgression events could be detected by intraspecific GWAS, with Gb_INT13 significantly improving fibre yield in *G. barbadense*. The present study describes the landscape of genetic introgression and selection between the two species, and highlights the genetic effects of introgression among populations, which can be used for future improvement of fibre yield and quality in *G. barbadense* and *G. hirsutum*, respectively*.*

## Significance Statement

*Gossypium hirsutum* and *Gossypium barbadense* are important species cultivated worldwide and the existence of introgression between the two species could have significant effects on agronomic traits. Introgressions tests, intraspecific selection analyses, phenotypic analysis and genome‐wide association studies were used to confirm the presence of introgression events and revealed the beneficial effect of introgression on fibre trait improvement during the parallel independent breeding of cotton varieties in Xinjiang, China*.*

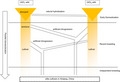

## INTRODUCTION

Six new world tetraploid cottons are derived from a single hybridisation between the A diploid and D diploid genome species (Wendel and Grover, 2015). Two of these species, *Gossypium hirsutum* and *Gossypium barbadense*, have been domesticated and cultivated in the world. Abundant germplasm resources for these species have been collected and applied globally (Campbell *et al.*, 2010). *G. hirsutum* (Upland cotton) is superior in terms of yield and accounts for more than 95% of world cotton production, whereas *G. barbadense* (Pima cotton) is superior for its fibre quality and accounts for about 2% of cotton production (Chen *et al.*, 2007). In addition, these two species differ with respect to other traits such as resistance to *Verticillium* wilt (Zhao *et al.*, 2018; Li *et al.*, 2019) and *Fusarium* wilt (Ulloa *et al.*, 2016). To take full advantage of natural genetic variations within gene pools, introducing beneficial alleles through interspecific hybridisation has been a strategy for broadening the genetic basis of cultivated cotton (Shim *et al.*, 2018). This strategy involves the invasion of a foreign genetic fragment into the host genome and is known as introgression.

During the modern breeding and research process, man‐made introgression lines derived from crosses between *G. hirsutum* and *G. barbadense* have improved fibre quality and resistance to certain pathogens (Fang *et al.*, 2014; Song *et al.*, 2017). In addition, natural and ancestral introgressions were reported between *G. hirsutum* and *G. barbadense* as early as 1990 and 1992, respectively (Percy and Wendel, 1990; Wendel *et al.*, 1992). Brubaker *et al.* (1993) documented asymmetry and bidirection in cytoplasmic introgression between *G. hirsutum* and *G. barbadense*. These introgression events have also been detected in modern cotton cultivars (Wang *et al.*, 2015; Fang *et al.*, 2017a). However, the genetic effects of introgression on agronomic traits have not been well studied in modern cotton cultivars. China is one of the major cotton producing countries. In China, varieties grown in early period (1940–1960) were directly introduced from foreign countries, whereas independent breeding started in 1960s. During this independent breeding era, man‐made crosses and selection have been a driving force for improving fibre quality and resistant traits by retaining favourable alleles of important loci. Thus, there is a need to understand the existence and genetic effects of selection and mutual introgression signatures in cultivars between or within *G. hirsutum* and *G. barbadense*.

The genetic study of populations is a main approach for analysing crop evolution and domestication (Schreiber *et al.*, 2018). The published cotton reference genomes of *G. hirsutum* and *G. barbadense* (Hu *et al.*, 2019; Wang *et al.*, 2019) have significantly contributed to our knowledge of cotton population genetics, and have allowed for the dissection of complex traits, in addition to uncovering domestication signatures and large‐scale genetic variations (Wang *et al.*, 2015; Wang *et al.*, 2017; Ma *et al.*, 2018; Wen *et al.*, 2018; Wen *et al.*, 2019). Introgression is common between species and is often related to adaptive traits (Whitney *et al.*, 2006; Clarkson *et al.*, 2014; Racimo *et al.*, 2015; Akpertey *et al.*, 2018). Detection of naturally mutual introgression is important for uncovering population history and structure (Reich *et al.*, 2009; Fontaine *et al.*, 2015) and multiple methods have been developed to achieve this, such as treemix software for inferring the population splits and mixtures (Pickrell and Pritchard, 2012; Karlsson *et al.*, 2014; Martin *et al.*, 2015; Pease and Hahn, 2015; Elworth *et al.*, 2018).

In the present study, we collected an interspecific panel consisting of 159 *G. hirsutum* and 70 *G. barbadense* cultivars developed in Xinjiang, the biggest cotton growing region in China, and the cultivars were planted in multiple environments to collect phenotypic data. We also included 30 *G. hirsutum* races (e.g. yucatanensis, richmondi, morrilli, etc.) in the study to determine (i) selection loci for fibre traits and a collinear relationship between *G. hirsutum* and *G. barbadense* cultivars; (ii) the existence and genomic location of introgression in modern cultivars; and (iii) the genetic and phenotypic effects of introgression loci on fibre traits during independent breeding in modern cultivars.

## RESULTS

### High‐density genetic variation map and population structure in the interspecific panel

**Figure 1:**
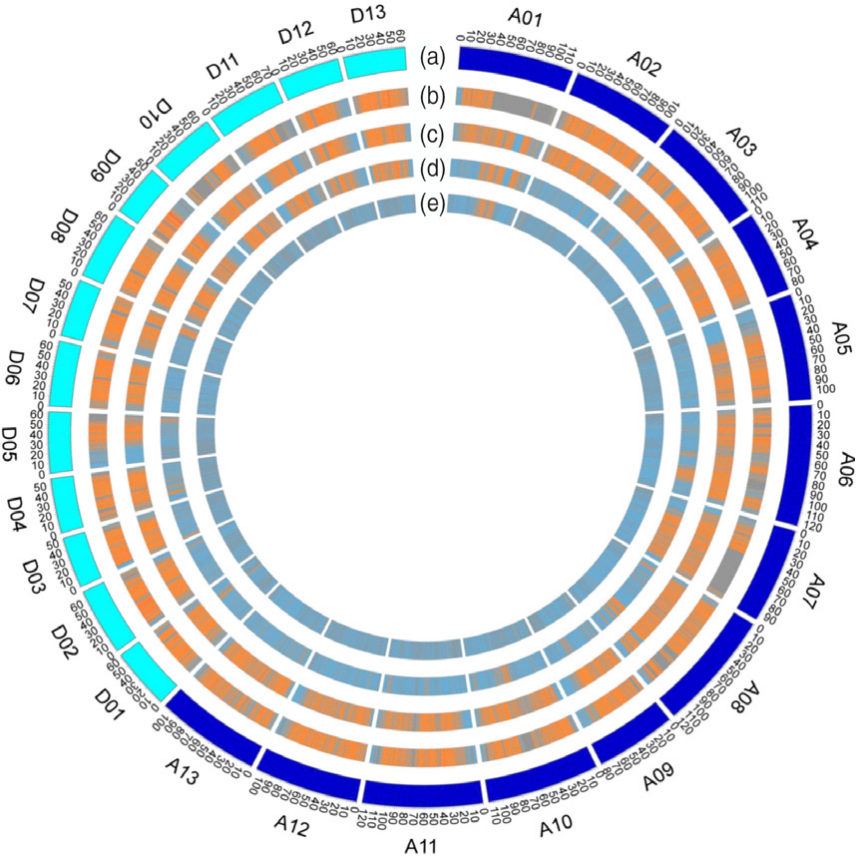
Genetic variation of single nucleotide polymorphisms (SNPs) between Gh and Gb. (a) The karyotype of *Gossypium hirsutum* reference genome. (b) The fixed interspecific SNPs between Gh and Gb. (c) The SNPs in Gh and Gb. (d) The SNPs in Gb. (e) The SNPs in Gh. Gh, *G. hirsutum* cultivars; Gb, *Gossypium barbadense* cultivars.

In the interspecific panel, 159 *G. hirsutum* cultivars (hereafter defined as Gh) and 70 *G. barbadense* cultivars (hereafter defined as Gb) from introduced and independent breeding cultivars in Xinjiang, China (Figure S1; Table S1) were re‐sequenced with 10× genomic coverage. Thirty *G. hirsutum* races (hereafter defined as Gh‐race) were collected from published data (Fang *et al.*, 2017a) (Table S1). A high‐density and ‐quality genetic variation map was then constructed from 6 318 993 single nucleotide polymorphisms (SNPs) in the interspecific panel, and a total of 1 034 682 SNPs in Gh, 1 507 705 SNPs in Gh and Gh‐race, and 3 465 402 fixed interspecific SNPs between Gh and Gb were identified (Table 1), which suggests extreme interspecific differentiation. The high‐density genetic variation map in four levels (fixed interspecific SNPs, Gh&Gb SNPs, Gb SNPs and Gh SNPs) indicated that the genetic variations were not uniformly distributed across chromosomes and regions (Figure 1). For example, the fixed interspecific SNPs on chromosome A01 showed the lowest density, whereas chromosome D02 showed the highest density (Figure 1b; Table 1).

**Table 1:** Genetic variation of single nucleotide polymorphisms (SNPs) in the interspecific panel

Chromosome	Length (kb)	Total SNPs	SNPs in Gh	SNPs in Gh and Gh‐race	Fixed interspecific SNPs	Interspecific SNP density (SNPs/kb)
A01	117 710.66	296 117	119 025	133 851	77 505	0.66
A02	108 049.53	334 016	43 867	66 812	208 185	1.93
A03	113 014.28	322 684	41 351	69 177	203 826	1.8
A04	85 114.396	256 939	29 867	55 084	155 254	1.82
A05	109 365.99	254 605	39 742	60 346	157 852	1.44
A06	124 007.24	366 059	73 070	98 789	150 953	1.22
A07	97 738.592	285 174	48 830	59 296	67 456	0.69
A08	122 327.75	356 600	70 894	84 854	168 524	1.38
A09	82 064.019	230 478	37 495	55 640	134 328	1.64
A10	114 802.33	313 878	50 092	81 309	166 584	1.45
A11	123 158.38	318 469	44 456	77 325	194 998	1.58
A12	107 624.58	287 341	37 590	59 800	190 402	1.77
A13	108 332.2	316 310	55 683	74 903	166 482	1.54
D01	63 183.132	181 762	33 054	47 034	107 888	1.71
D02	69 812.089	234 694	35 881	52 992	154 738	2.22
D03	52 678.327	168 557	17 462	31 086	107 436	2.04
D04	56 408.347	184 376	17 433	31 764	100 221	1.78
D05	62 903.527	154 990	28 983	38 612	81 017	1.29
D06	66 842.384	197 091	33 124	46 548	130 982	1.96
D07	59 231.761	162 121	25 101	38 677	107 492	1.81
D08	69 011.085	206 546	28 929	44 368	121 898	1.77
D09	52 796.336	153 394	26 935	35 000	92 159	1.75
D10	67 976.702	200 943	29 394	50 176	67 436	0.99
D11	72 910.981	171 376	21 730	38 942	103 533	1.42
D12	62 667.218	171 855	23 674	35 169	115 807	1.85
D13	63 316.526	192 618	21 020	40 151	132 446	2.09
At	141 3309.9	3 938 670	691 962	977 186	2 042 349	1.46
Dt	819 738.42	2 380 323	342 720	530 519	1 423 053	1.74
Total	2 233 048.4	6 318 993	1 034 682	1 507 705	3 465 402	1.6

**Figure 2:**
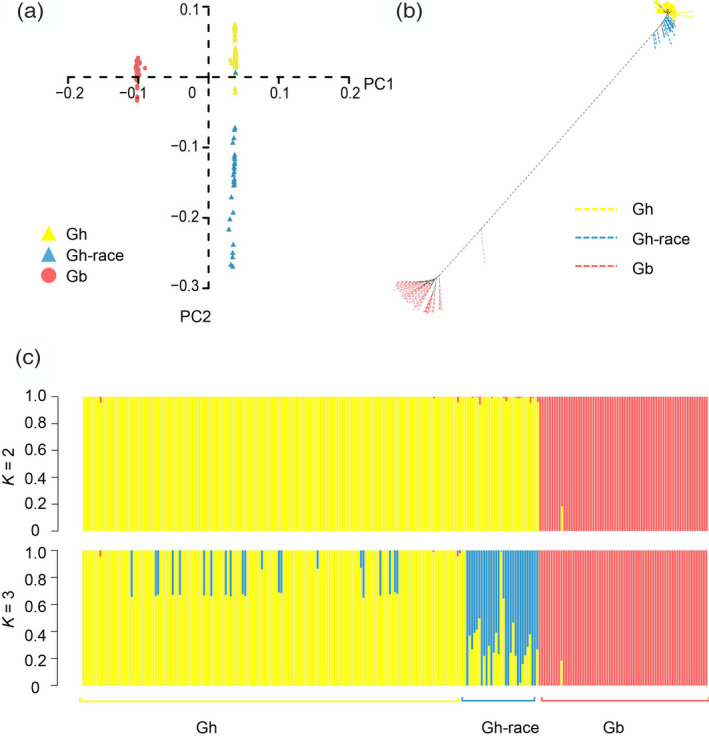
Population structure of the interspecific panel. (a) Principal component analysis of 259 cotton accessions. (b) Phylogenetic tree of 259 cotton accessions. (c) Population structure of 259 cotton accessions when *K* (number of groups) = 2 and *K* = 3. Gh, *Gossypium hirsutum* cultivars; Gb, *Gossypium barbadense* cultivars; Gh‐race, *G. hirsutum* races.

To demonstrate genetic relationships among three groups of cotton accessions (Gh, Gb and Gh‐race) (Table S1), population structure analyses were performed. Principal component analysis showed that the first eigenvector (PC1) clearly separated Gb from Gh and Gh‐race, while the second eigenvector (PC2) distinguished Gh from Gh‐race (Figure 2a). Two distinct branches of the phylogenetic tree support the hypothesis that *G. hirsutum* and *G. barbadense* species have been remarkably diversified over the course of evolutionary history (Figure 2b). The population structure collaboratively indicated that a significant population stratification exists between *G. hirsutum* and G. *barbadense* species (*K* = 2), and the Gh, Gh‐race and Gb groups were generally distinguished from each other (*K* = 3) (Figure 2c). Interestingly, Figure 2(c) shows that the population structure between *G. hirsutum* and *G. barbadense* species is slightly mixed, which may be a result of introgression.

### Estimation of interspecific differentiation and introgression

**Figure 3:**
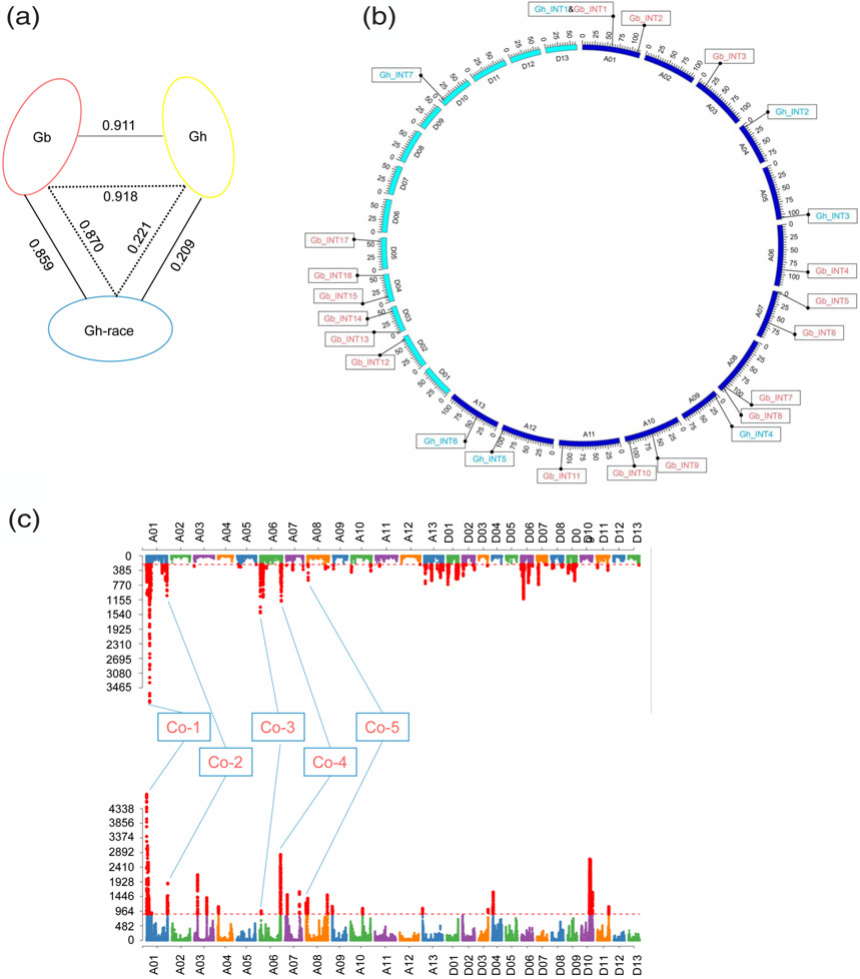
Population differentiation, introgression and selection. (a) The population differentiation with and without introgression events among Gh, Gb and Gh‐race groups. The solid lines represent the groups with introgression loci; the dotted lines represent groups without introgression loci. Gh, *Gossypium hirsutum* cultivars; Gb, *Gossypium barbadense* cultivars; Gh‐race, *G. hirsutum* races. (b) Genetic introgressions in genome‐wide scale. Gb‐INT, introgression event from Gh to Gb; Gh‐INT, introgression event from Gb to Gh. (c) Selective sweeps and co‐selected loci in Gh and Gb panels.

To explain the uneven distribution of SNPs in the interspecific panel (Figure 1) and precisely estimate interspecific differentiation, genome‐wide population divergence with Gb, Gh and Gh‐race groups was tested. The fixation index (*F*_ST_) showed that a notable interspecific differentiation (*F*_ST_ = 0.911) existed between the Gb and Gh groups, whereas the intraspecific differentiation (*F*_ST_ = 0.209) between Gh and Gh‐race groups was lower. A lower interspecific differentiation between Gb and Gh‐race (*F*_ST_ = 0.859) was also observed compared to Gb and Gh differentiation (*F*_ST_ = 0.911) (Figure 3a). Phenotypic data in multiple environments also collectively revealed a significant interspecific difference (Table S2) for nine fibre traits (LP, lint percentage; SCW, seed cotton weight; LW, lint weight; FL, fibre length; FS, fibre strength; MV, micronaire value; FU, fibre uniformity; SFC, short fibre content; FE, fibre elongation) in six environments. Analysing with BLUP (Best Linear Unbiased Prediction) phenotype data revealed that the Gh group had a significantly higher fibre yield (*P* < 0.01) compared to the Gb group in traits of LP, SCW and LW; however, the Gb group had significantly better fibre quality (*P* < 0.01) than the Gh group in traits of FL, FS, FU and SFC (Figure S2). In general, the correlation and heritability of most fibre traits showed a higher value in the Gh and Gb panel than in the Gh or Gb panel and this may have resulted from the dramatic interspecific difference of phenotype; in the Gh and Gb panels, the correlation indicated that FL remains stable in different environments and FL has a higher heritability, whereas LP has a relative low correlation and heritability (Table S3). The fixation index at the chromosome level revealed an accurate intraspecific and interspecific differentiation map (Figure S3). Additionally, most SNPs harboured a high *F*_ST_ value between Gh and Gb, although abundant loci with relatively lower differentiation also existed on the chromosomes (Figure S3b).

To determine whether the lower differentiation loci in the interspecific map belonged to introgressed or undifferentiated loci, ‘three population statistics’ and phylogenetic distance analysis were performed using a 1‐Mb chromosome scale. The results showed that history introgression events occurred between the Gh and Gb groups (*f*_3_ = −0.006 and *Z* score = −73.3585) (Table S4), providing evidence that interspecific introgression has been retained in modern breeding cultivars. A genome‐wide scan of introgression with a phylogenetic tree analysis indicated that a total of seven introgression events, including 10 introgression loci, were detected in *G. hirsutum* cultivars. In addition, 17 introgression events, including 32 introgression loci, were detected in *G. barbadense* cultivars (Figure 3b; Table S5), which demonstrates that asymmetrical introgression events flowed more frequently from *G. hirsutum* to *G. barbadense* than from *G. barbadense* to *G. hirsutum*. The phylogenetic trees can clearly distinguish the non‐introgression (Figure S4a) or introgression region (Figure S4b) between Gh and Gb, and it can be observed that Gb accessions were mixed in Gh branch in A06~87 Mb (Figure S4b). Interestingly, a bidirectional introgression locus (Gh_INT1 and Gb_INT1) was found on chromosome A01 (Figure 3b), indicating that *G. hirsutum* and *G. barbadense* cultivars mixed in both branches of the phylogenetic tree (Figure S4c). This bidirectional introgression region showed a lower population differentiation (Figure S3b). In the Gh_INT1 event, differentiation tests between introgression and non‐introgression groups showed that one stable effective locus existed in this region; for Gb_INT1, differentiation tests showed that three stable effective loci existed in this region (Table S6). Introgression analyses indicated that 87 of 159 *G. hirsutum* cultivars harboured genomic fragments from *G. barbadense* species, although all 70 *G. barbadense* cultivars harboured uneven numbers of genomic fragments from *G. hirsutum* species. Both core Gh breeding parent cultivars (e.g. Y‐231 and Y‐232) and core Gb breeding parent cultivars (e.g. Y‐217, Y‐218 and Y‐219) had introgression events, which suggests that most of introgression events occurred before the Xinjiang independent breeding era. Interestingly, the fixed value (*F*_ST_) between Gh and Gb was increased after excluding the genetic introgression loci (Figure 3a), suggesting that introgression decreased population divergence.

To determine whether introgression has a genetic effect on the nine fibre traits, we performed linear analysis between introgression loci and these traits in multiple environments. The results showed that accumulated introgression loci could generally improve fibre traits for both the Gh and Gb panels (Figures S5 and S6). In the Gh panel, SCW, LW, FU in E1, FS and FE in E2, SCW, LW and FL in E3 and LW in E5 and E6 had a significant correlation between introgression loci and fibre traits (*P* < 0.05); for the Gb panel, SCW, LW and FS in E1, LP in E5 and FE in E6 had a significant correlation between introgression loci and fibre traits (*P* < 0.05). Remarkably, when nine fibre traits in six environments were compared between introgression and non‐introgression groups in each introgression locus, three stable effective loci were detected in the Gh panel and 17 stable effective loci were detected in the Gb panel. Notably, the introgression group harboured a higher significant value of fibre traits than the non‐introgression group in abundant introgression loci (Table S6). Hence, the benefit of introgression events has been retained in breeding populations to improve fibre traits.

### Genetic introgression signature during intraspecific selection as revealed by high‐density genetic variation maps and genome‐wide association studies (GWAS)

We utilised multiple bioinformatics software to construct three high‐density maps with three kinds of genetic variations, SNP, insertion‐deletion (Indel) and structural variation (SV) based on re‐sequencing data of Xinjiang cultivars. In total, 3 876 899 SNPs, 756 666 indels and 39 363 SVs were detected in the Gh panel (Table S7; Figures S7a, S8a and S9a) and 2 972 892 SNPs, 596 859 indels and 20 588 SVs were detected in the Gb panel (Table S8; Figures S7b, S8b and S9b). Three maps of genetic variations collectively revealed that introgression loci had more genetic variation and diversity (pi) for both Gh and Gb panels (Tables S7 and S8). In particular, this phenomenon was obvious on chromosome A01, which included a long bidirectional introgression fragment (Figures S7–S9). Therefore, introgression events significantly increased genetic variation and diversity in intraspecific populations.

During introduction and independent breeding, genomic loci associated with important agronomic traits were selected and showed an unbalanced allele frequency in the population. In this study, 52 selective sweep loci were identified in the Gh panel, as well as 19 selective sweep loci in the Gb panel (Figure 3c; Table S9). Collinear analysis between the interspecific selective sweep loci, which were aligned to the *G. hirsutum* reference genome, identified five co‐selected loci between the Gh and Gb panels (Figure 3c). In comparison with the published quantitative trait locus (QTL) by GWAS (Wang *et al.*, 2017; Fang *et al.*, 2017b; Ma *et al.*, 2018), 66 reported QTL overlapped in 16 selective sweeps for the Gh panel (Table S10). These results indicate that the independent breeding cultivars in Xinjiang have retained an introgression signature.

Additionally, we performed GWAS of nine fibre traits with *G. hirsutum* and *G. barbadense* populations, and estimated linkage disequilibrium (LD) for association mapping resolution (Table S11; Figures S10 and S11). We collected phenotype data of nine fibre traits from six environments, predicted one BLUP phenotype data and performed genome‐wide association analysis with the intraspecific Gh and Gb panels. Association mapping revealed a total of 40 and 63 QTL in Gh and Gb, respectively (Tables S12 and S13; Figures S12 and S13). Within the associated QTL, we found that *q‐A01‐FE* overlapped in Gb_INT1; *q‐A08‐FU‐2* overlapped in Gb_INT8; and *q‐D05‐SFC* overlapped in *Gb_INT17*; importantly, two QTL, *q‐D03‐SCW* and *q‐D03‐LW* (Figure S13a,b), which were located in the region of introgression event Gb_INT13 (Table S13), also significantly affected fibre quality traits (e.g. FL, FS and FU) (*P* < 0.05) (Table S6).

### Gene expression pattern in introgression QTL *q‐D03‐SCW* and *q‐D03‐LW* is illustrated by a chromosome segment substitution line (CSSL)

**Figure 4:**
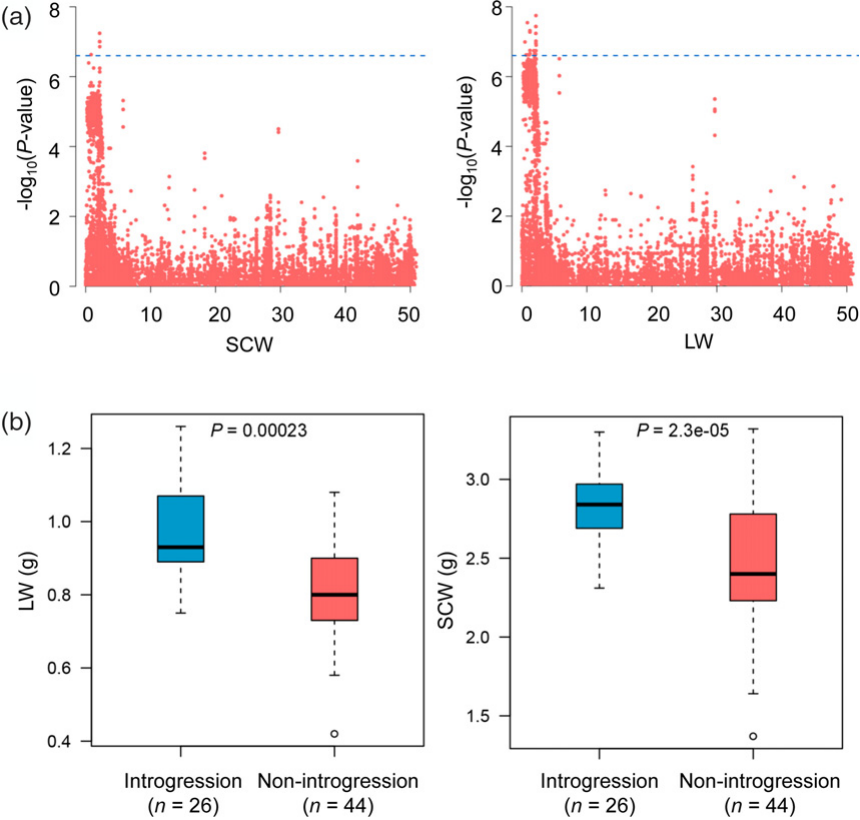
The fibre yield quantitative trait locus introgressed from Gh to Gb. (a) Genome‐wide associated locus of fibre yield traits in Gb on chromosome D03. (b) The genetic effects of two fibre yield traits between the introgression and non‐introgression groups in the Gb panel. SCW, seed cotton weight; LW, lint weight; Gh, *Gossypium hirsutum* cultivars; Gb, *Gossypium barbadense* cultivars.

**Figure 5:**
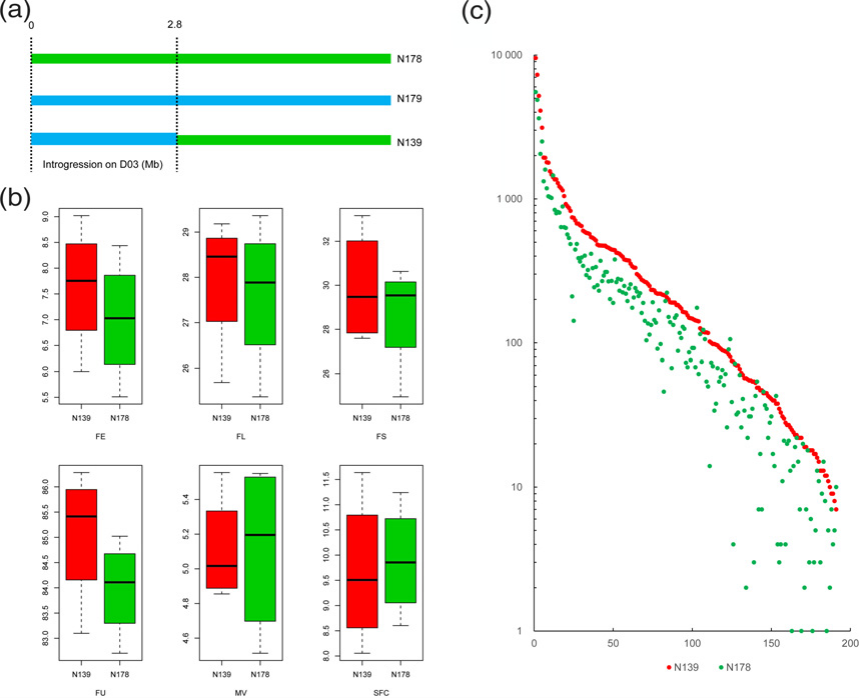
Fibre traits and gene expressions of N139 and N178 in the introgression locus. (a) Introgression diagram of N139 compared with N178 and N179 on chromosome D03. (b) Box‐plot of six fibre traits in N139 and N178. (c) Gene expressions of N139 and N178 in the introgression locus. N139, a chromosome segment substitution line (CSSL); N178, *Gossypium hirsutum* cv. E22; N179, *Gossypium barbadense* acc. 3‐79; FE, fibre elongation; FL, fibre length; FS, fibre strength; FU, fibre uniformity; MV, micronaire value; SFC, short fibre content.

A Manhattan plot showed that SNPs were significantly associated with the yield traits SCW and LW on chromosome D03 [−log_10_(*P*‐value) > 6.6] (Figure 4a). These SNPs overlapped with the Gb_INT13 introgression, and a long segment of LD block was identified in this region (Figure S14). Compared with the non‐introgression group, the introgression group harboured traits favouring a higher yield (Figure 4b). Furthermore, we found an introgression line, N139, which has been reported to harbour an introgression locus (D03: 0–2.8 Mb in *G. hirsutum*) from *G. barbadense* (Figure 5a) (Wang *et al.*, 2019). An analysis of fibre traits revealed that the introgression line showed a better fibre quality compared to that of its parent (N178, *G. hirsutum* cv. E22), indicating that this region was also beneficial for improving fibre traits in the genetic background of *G. hirsutum* (Figure 5b). Gene expression data for the introgression locus indicated that 191 genes were preferentially expressed in 10‐day post‐anthesis fibre, and these genes showed a higher expression in the CSSL (N139) compared to N178 (Figure 5c). Therefore, an introgression event may change expression patterns in fibre tissues.

## DISCUSSION

### Dramatic diversification exists between *G. hirsutum* and *G. barbadense* cultivars during parallel breeding

The present study reflects dramatic genetic and phenotypic diversification between *G. hirsutum* and *G. barbadense* cultivars (Figures 1 and 3a; Figure S2). As reported, these two cultivated species, *G. hirsutum* and *G. barbadense*, have a short evolutionary history of approximately 1–2 million years (Wendel and Grover, 2015). A selective sweep has been shown to strengthen subspecies differentiation between *japonica* and *indica* rice cultivars (Yuan *et al.*, 2017); we found that the independent breeding of *G. hirsutum* and *G. barbadense* cultivars also strengthened population diversification of interspecific cultivars (Figure 3a). In terms of population structure, Gh and Gb were clustered, respectively, whereas Gh and Gh‐race groups were in a continue distribution, which is consistent with the domestication of *G. hirsutum* process. One expected Gb dot was located away from the Gb cluster (Figure 2a,b), which may be attributed to the complex and obscure origin of *G. barbadense* cultivars. Southern Mexico and Central America are assumed to be the regions of origin for *G. hirsutum*, whereas the Andean region of Peru, Ecuador and Colombia are the assumed regions of origin for *G. barbadense* (Richmond, 1951). An independent domestication of approximately 5000 years has also been predicted (Brubaker and Wendel, 1994; Westengen *et al.*, 2005; Gross and Olsen, 2010), especially for *G. hirsutum*, which spans the wild‐to‐domesticated continuum (wild, dooryard, land race and cultivated types) (Rapp *et al.*, 2010). The combined factors of natural mutation, selection and human domestication may drive the divergence and evolution of these two species (Figure 3).

Because of the highly homologous relationship between *G. hirsutum* and *G. barbadense* (Wang *et al.*, 2019), genomic loci containing functional genes associated with agronomic traits may undergo parallel domestication and selection (Wendel and Grover, 2015). Presently, few studies have demonstrated the co‐domestication and selection phenomenon between *G. hirsutum* and *G. barbadense*. Domestication and selection are common in plants (e.g. rice, tomato and barely), and positive selection rates can be approximately 7.6% in maize (Hufford *et al.*, 2012). In the present study, of the 52 and 19 selective loci identified in *G. hirsutum* and *G. barbadense* cultivars, respectively, only five were found to be co‐selected (Figure 3c; Table S9). This finding suggests that the breeding of *G. hirsutum* and *G. barbadense* belongs to an independent process, whereas the co‐selected regions may result from a co‐linear functional genomic relationship between the cotton species. The co‐selected loci in the selection map also showed a greater amount of selection in the At subgenome than in the Dt subgenome, which is consistent with asymmetric selection in *G. hirsutum* populations (Wang *et al.*, 2017).

### Asymmetric mutual introgression exists between *G. hirsutum* and *G. barbadense* cultivars

The two globally distributed cultivated cottons, *G. hirsutum* and *G. barbadense*, could be crossed without a reproductive barrier, which may be an important factor for inducing interspecific introgressions. The availability of a high‐quality reference genome allowed us to detect historical introgression. Genetic analyses, including population structure (Figure 2), population divergence (Figure S3) and introgression tests (Tables S4 and S5), collectively showed that interspecific introgression exists in cotton. The *F*_ST_ indicated a lower differentiation value in the introgression loci; however, a number of lower differentiation regions existed without significant signature of an introgression event (Figure S3). These regions may result from the complex of a polyploid cotton genome or less divergence existed between the *G. hirsutum* and *G. barbadense* genomes in history. Therefore, these regions were not inferred from the phylogenetic tree, and a number of introgressions may have not been detected as a result of the limited scanning window. These ancestral introgressions may act in a role opposite to that of reproductive isolation; alternatively, regions with lower interspecific differentiation may exist at a genome‐wide level. As early as 1994, bidirectional nuclear introgressions between *G. hirsutum* and *G. barbadense* were detected in an asymmetrical distribution (Brubaker and Wendel, 1994). The present study identified 17 introgression events that flowed from Gh to Gb, wheeras only seven introgression events flowed from Gb to Gh, which is consistent with previous studies (Brubaker *et al.*, 1993).

Potential mechanisms may exist in this asymmetrical introgression phenomenon. Introgression is commonplace in animals and plants and is related to environmental adaptation and trait improvement (Lucek *et al.*, 2014; Ai *et al.*, 2015; Clark *et al.*, 2015; Zhang *et al.*, 2016; Zou *et al.*, 2019). Therefore, we hypothesised that the asymmetrical introgression mechanism in cotton species may relate to adaptability. In comparison with that of *G. hirsutum*, the *G. barbadense* cytoplasm background may present a better compatibility, although no reproduction barrier existed between *G. hirsutum* and *G. barbadense*. A previous study reported that different maternal cytoplasmic environments (*G. hirsutum* and *G. barbadense*) have significant effects on reproductive traits such as infertility and seed production (Dai *et al.*, 2016); on the other hand, *G. hirsutum* displays a better yield and adaption and is cultivated worldwide, which may assist transfer of the *G. hirsutum* nuclear genome to *G. barbadense*. Fang *et al.* (2017a) also found that the genes in the introgression events were enriched in the reproduction, epithelial cell development and cell proliferation processes. Therefore, the asymmetrical introgression between *G. hirsutum* and *G. barbadense* may result from these two aspects.

There is strong evidence to suggest that introgression occurred in the early breeding era (Richmond, 1951; Wang *et al.*, 1995). We show here that introgression loci existed in both core parents introduced from foreign countries and breeding cultivars, whereas no introgression events occurred during the independent breeding of Xinjiang elite cultivars. Taken together, these results suggest that the retained introgression event in modern cultivars may derive from the early breeding era. At present, introgression lines with single or very few genomic fragments have been developed to improve single traits. However, the present study indicates that a better strategy for improving modern cultivars involves the introduction of multiple beneficial introgression loci.

### The introgressions produce significant genetic and phenotypic effects

In the present study, bidirectional introgression events enriched the density of SNPs, indels and SVs within intraspecific populations (Figures S7 and S8). This broadens the gene pools and also the functional diversity in *G. hirsutum* and *G. barbadense*. Artificial introgression lines have been developed and studied with agronomic traits in rice (Ma *et al.*, 2016), maize (Liu *et al.*, 2016), wheat (Ali *et al.*, 2016) and cotton (Wang *et al.*, 2019). In the present study, the relationship between the number of introgression loci and fibre traits indicated that introgressions are beneficial for the improvement of fibre traits within intraspecific species (Figures S5 and S6). A Student's *t*‐test conducted between introgression and non‐introgression groups showed three stable effective loci in the Gh panel and 17 stable effective loci in the Gb panel (Table S6). Certain loci have less of an effect on fibre traits, and these loci may instead be important for resistance to diseases or for other traits. Furthermore, the phenotypic effects of three introgression events could be detected by GWAS, and Gb_INT13 significantly improved fibre yield in *G. barbadense* (Figure 4). Intriguingly, introgression in a CSSL with the same locus in both *G. barbadense* and *G. hirsutum* affected fibre characteristics and also modified the gene expression pattern, indicating transgressive gene expression in this region (Figure 5). The transgressive expression model has also been found in hybrid sunflower species (Lai *et al.*, 2006). This suggests that exploiting and applying transgressive genetic effect loci with respect to interspecific introgression lines is important for future breeding programmes.

In summary, beneficial reciprocal genetic introgression events in *G. hirsutum* and *G. barbadense* cultivars are retained from the evolution and independent breeding of cotton. Using a high‐density interspecific SNP map and three types of genetic variation maps of intraspecific species for *G. hirsutum* and *G. barbadense* cultivars, we identified 52 *G. hirsutum* and 19 *G. barbadense* selective sweep loci in the population, as well as five co‐selected loci in both populations. We also uncovered asymmetrical interspecific introgression between *G. hirsutum* and *G. barbadense*; 17 interspecific introgression events occurred in *G. barbadense*, whereas seven events occurred in *G. hirsutum*. We fine‐mapped introgression loci with significant effects on fibre traits and, in particular, an important introgression event, Gb_INT13, was identified with the GWAS method, and we explored its transgressive expression pattern with a CSSL. The findings of the present study should increase our understanding of genetic introgression and help to advance interspecific molecular breeding.

## EXPERIMENTAL PROCEDURES

### Materials and phenotype evaluation

To evaluate the fibre traits, 159 *G. hirsutum* cultivars (Gh) and 70 *G. barbadense* cultivars (Gb) were planted with two replicates in multiple environments including E1 (Shihezi, Xinjiang, China, in 2016; E85.94°, N44.27°); E2 (Kuerle, Xinjiang, China, in 2016; E86.06°, N35.05°); E3 (Shihezi, 2017); E4 (Kuerle, 2017); E5 (Shihezi, 2018); and E6 (Kuerle, 2018). One BLUP (Best Linear Unbiased Prediction) phenotype data were predicted from the phenotype data of six environments by a linear model, and the r package (lme4) (R Foundation for Statistical Computing, Vienna, Austria) was applied to calculate the BLUP phenotype data in this model. The mature fibre was collected from the respective environments and, after ginning, yield traits were evaluated, including seed cotton weight (SCW), lint weight (LW) and lint percentage (LP); fibre quality traits including upper half mean length (FL), fibre strength (FS), micronaire value (MV), fibre unity (FU), short fibre content (SFC) and fibre elongation (FE) were measured using a HVI1000 (User Technologies, Inc., Uster, Switzerland) under conditions of 20°C and 65% relative humidity.

### Population re‐sequencing and genetic variation calling

For each *G. hirsutum* and *G. barbadense* cultivar, a young leaf was collected from the plant, and genomic DNA was extracted for construction of a paired‐end sequencing library to perform 10× genomic coverage re‐sequencing with the HiSeq 2000 platform (Illumina, Inc., San Diego, CA, USA). The clean genomic data were generated and deposited in NCBI Sequence Read Archive (SRA) (https://www.ncbi.nlm.nih.gov/sra) under accession number PRJNA473334. Thirty Gh‐race accessions were downloaded from the NCBI databank (https://www.ncbi.nlm.nih.gov) under accession numner PRJNA257154 (Table S1).

The clean reads derived from 159 *G. hirsutum* cultivars, 70 *G. barbadense* cultivars and 30 Gh‐race accessions were aligned against the *G. hirsutum* reference genome TM‐1 (Wang *et al.*, 2019). Notably, the 70 *G. barbadense* cultivars were also aligned against the *G. barbadense* reference genome 3–79 (Wang *et al.*, 2019) using bwa, version 0.7.10 (Li and Durbin, 2009). The alignments were sorted and processed with picard, version 1.112 (https://broadinstitute.github.io/picard).

The processed Binary Alignment/Map (BAM) files were applied to call three types of genetic variations, including SNPs, Indels and SVs. The HaplotypeCaller module of gatk, version 3.1.1 (McKenna *et al.*, 2010) was applied to produce GVCF files of each accession; GenotypeGVCFs module in the GATK toolkit was applied to merge all individual GVCF files together, and genetic variations of SNPs and Indels were obtained in intraspecific (Gh or Gb) or interspecific panels (Gh*,* Gb and Gh‐race). bcftools, version 1.3 (Li *et al.*, 2009) was applied to filter the SNPs with parameters QD < 2.0 || MQ < 40.0 || FS> 60.0 || MQRankSum < −12.5 || ReadPosRankSum < −8.0, and the Indels were filtered with parameters QD < 2.0 || FS> 200.0 || ReadPosRankSum < −20.0. beagle, version 4.1 (Browning and Browning, 2007) was applied to impute the missing genotype. lumpy, version 0.2.13 (Layer *et al.*, 2014) was applied to call the SVs with the BAM file, and then genotyping was performed with SVTyper (https://github.com/hall‐lab/svtyper). vcftools, version 0.1.14 (Danecek *et al.*, 2011) was applied to filter the genotype data with parameter MinQ > 200 and merge the SVs of all the intraspecific individuals together. The genetic variation maps were drawn using the r package (CMplot).

### Population structure, introgression and differentiation analyses

In total, 6 318 993 high quality SNPs in the interspecific species panel were applied to conduct the population structure analysis and perform a genome‐wide scanning of the interspecific population introgression. admixture, version 1.3 (Alexander *et al.*, 2009) was applied to impute the population structure number from 1 to 9, and tassel, version 5.0 (Bradbury *et al.*, 2007) was used to perform principal component analysis and phylogenetic analysis. Based on the allele frequency in the interspecific species panel, the fixed species‐specific SNPs (Gh alternative allele < 6 and Gb alternative allele > 134) were quantified using vcftools, version 0.1.14.

The introgression between three population groups was detected by *f_3_* statistic of treemix, version 1.13 (Pickrell and Pritchard, 2012). To reveal fine‐scaled introgression loci and introgression materials, a phylogenetic tree was imputed by SNPs in a 1‐Mb sliding window scale using tassel, version 5.0 and itol (https://itol.embl.de) was applied to draw the phylogenetic tree. To quantify the number of introgression cultivars, we designated only the significant divergent region as the introgression locus, although Gb and Gh were mixed together in phylogenetic tree.

The population differentiation among Gb, Gh and Gh‐race was calculated by vcftools (parameter: ‐‐fst‐window‐size 2 000 000 ‐‐fst‐window‐step 50 000); to explore the introgression effect on population differentiation, we excluded the SNPs located in the introgression region, and calculated the population differentiation with the same parameter by vcftools again. The scatter plot map of population differentiation was plotted using circos, version 0.67 (Krzywinski *et al.*, 2009).

### Genome‐wide selective sweeps and association analyses in two intraspecific populations

The high‐density SNPs within intraspecific populations (Gh and Gb) were applied to impute the selective sweeps using sweed, version 3.2.1 (Nielsen *et al.*, 2005). Depending on the domestication and selection of crop genomes, the top 5% value of the composite likelihood value of the SNPs was set as the selective sweep SNPs. The threshold values of Gh and GB were therefore set as 228 and 869, respectively. The selective sweep SNPs located within the distance of LD decay were set as the same select sweep locus.

High‐quality SNPs (minor allele frequency > 0.05) of intraspecific populations (Gh and Gb) were applied to associate with nine fibre traits (SCW, LW, LP, FL, FS, MV, FU, SFC and FE) in multiple environments. The association mapping was performed with the LMM model of gemma, version 0.97 (Zhou and Stephens, 2012). The threshold of respective panels was determined by the significant *P* value, which was calculated using gec, version 0.2 (Li *et al.*, 2012).

### RNA‐sequencing data analyses of a chromosome segment substitution line (CSSL)

The RNA‐sequencing data of 10‐day post‐anthesis fibres of a CSSL (N139) and its parents (*G. hirsutum* cv. E22, N178; *G. barbadense* acc. 3‐79, N179) was downloaded from PRJNA433615 in the NCBI databank (Wang *et al.*, 2019). tophat, version 2.0.13 (Trapnell *et al.*, 2009) was applied to align the clean reads to the TM‐1 reference genome (Wang *et al.*, 2019) and htseq, version 0.8.0 (Anders *et al.*, 2015) was used to calculate gene expression. Six fibre traits (FL, FS, MV, FU, SFC and FE) of N139, N178 and N179 were collected from previously published phenotype data (Wang *et al.*, 2019).

## CONFLICT OF INTERESTS

The authors declare that they have no conflicts of interest.

## AUTHOR CONTRIBUTIONS

ZL and CY designed and supervised the research. XD and ZL revised the manuscript. PS, BT, GN, XN, CY and YY investigated phenotypic traits in Kuerle and Shihezi. TW performed genotypic and bioinformatic analyses. TW and XN wrote the main manuscript text. All authors reviewed the final manuscript submitted for publication.

## Supporting information


**Figure S1.** The introduction and independent breeding of cultivars in Xinjiang, China. (a) The architecture of *Gossypium hirsutum* and *Gossypium barbadense* cultivars in Xinjiang. (b) Introduction and independent breeding in Xinjiang.
**Figure S2.** BLUP phenotype data of nine fibre traits between Gb and Gh groups. LP, lint percentage; SCW, seed cotton weight; LW, lint weight; FL, fibre length; FS, fibre strength; MV, micronaire value; FU, fibre uniformity; SFC, short fibre content; FE, fibre elongation.
**Figure S3.** Population divergence between Gh, Gb and Gh‐race groups. (a) Karyotype of the *G. hirsutum* reference genome. (b) Population divergence between Gh and Gb. (c) Population divergence between Gh and Gh‐race. Gh, *Gossypium hirsutum* cultivars; Gb, *Gossypium barbadense* cultivars; Gh‐race, *G. hirsutum* races.
**Figure S4.** Phylogenetic tree based on genetic variations in the A06~3 Mb (a), A06~87 Mb (b), and A01~50 Mb (c). Gh, *Gossypium hirsutum* cultivars; Gb, *Gossypium barbadense* cultivars; Gh‐race, *G. hirsutum* races.
**Figure S5.** The collinear relationship between nine fibre traits and number of introgression loci in the Gh panel. The relationship in E1 (a), E2 (b), E3 (c), E4 (d), E5 (e) and E6 (f). LP, lint percentage; SCW, seed cotton weight; LW, lint weight; FL, fibre length; FS, fibre strength; MV, micronaire value; FU, fibre uniformity; SFC, short fibre content; FE, fibre elongation; Gh, *Gossypium hirsutum* cultivars.
**Figure S6.** The collinear relationship between nine fibre traits and the number of introgression loci in the Gb panel. The relationship in E1 (a), E2 (b), E3 (c), E4 (d), E5 (e) and E6 (f). LP, lint percentage; SCW, seed cotton weight; LW, lint weight; FL, fibre length; FS, fibre strength; MV, micronaire value; FU, fibre uniformity; SFC, short fibre content; FE, fibre elongation; Gb, *Gossypium barbadense* cultivars.
**Figure S7.** Genome‐wide single nucleotide polymorphisms (SNPs) in the Gh and Gb panels. Genome‐wide SNPs in the Gh panel (a) and the Gb panel (b). The number of SNPs was within a 1‐Mb window size. Gh, *Gossypium hirsutum* cultivars; Gb, *Gossypium barbadense* cultivars; the red box indicates bidirectional introgression on chromosome A01.
**Figure S8.** Genome‐wide insertion‐deletions (Indels) in the Gh and Gb panels. Genome‐wide Indels in the Gh panel (a) and the Gb panel (b). The number of Indels was within a 1‐Mb window size. Gh, *Gossypium hirsutum* cultivars; Gb, *Gossypium barbadense* cultivars; the red box indicates bidirectional introgression on chromosome A01.
**Figure S9.** Genome‐wide structure variations (SVs) in the Gh and Gb panels. Genome‐wide SVs in the Gh panel (a) and the Gb panel (b). The number of SVs was within a 0.1‐Mb window size. Gh, *Gossypium hirsutum* cultivars; Gb, *Gossypium barbadense* cultivars; the red box indicates bidirectional introgression on chromosome A01.
**Figure S10.** Linkage disequilibrium (LD) of At subgenome in the Gh and Gb panels. LD of At subgenome in the Gh panel (a) and the Gb panel (b). Gh, *Gossypium hirsutum* cultivars; Gb, *Gossypium barbadense* cultivars.
**Figure S11.** Linkage disequilibrium (LD) of Dt subgenome in the Gh and Gb panels. LD of Dt subgenome in the Gh panel (a) and the Gb panel (b). Gh, *Gossypium hirsutum* cultivars; Gb, *Gossypium barbadense* cultivars.
**Figure S12.** Manhattan plots of genome‐wide association in the Gh panel in different environments. Manhattan plots of FL in E1 (a), FS in E1 (b), FU in E1 (c), LP in E1 (d), LW in E1 (e), MV in E1 (f), SCW in E1 (g), SFC in E1 (h), FL in E2 (i), FS in E2 (j), FU in E2 (k), LP in E2 (l), LW in E2 (m), MV in E2 (n), SCW in E2 (o), FL in BLUP (p), FS in BLUP (q) and SFC in BLUP (r). LP, lint percentage; SCW, seed cotton weight; LW, lint weight; FL, fibre length; FS, fibre strength; MV, micronaire value; FU, fibre uniformity; SFC, short fibre content; FE, fibre elongation; Gh, *Gossypium hirsutum* cultivars.
**Figure S13.** Manhattan plots of genome‐wide association in the Gb panel in different environments. Manhattan plots of SCW in E1 (a), LW in E1 (b), SFC in E1 (c), FU in E1 (d), LW in E2 (e), SCW in E2 (f), LP in E2 (g), FU in E2 (h), MV in E2 (i), SCW in E3 (j), LP in E3 (k), FL in E3 (l), FS in E3 (m), FU in E3 (n), FE in E3 (o), FU in E4 (p), FL in E4 (q), FE in E4 (r), FS in E4 (s), FE in BLUP (t), FL in BLUP (u), FS in BLUP (v) and SFC in BLUP(w). LP, lint percentage; SCW, seed cotton weight; LW, lint weight; FL, fibre length; FS, fibre strength; MV, micronaire value; FU, fibre uniformity; SFC, short fibre content; FE, fibre elongation; Gb, *Gossypium barbadense* cultivars.
**Figure S14.** Linkage disequilibrium in the introgression event of Gb_INT13. The red triangle indicates the LD block. Gh, *Gossypium hirsutum* cultivars; Gb, *Gossypium barbadense* cultivars.

**Table S1.** Information on the collected germplasm resources.
**Table S2.** Statistics of nine fibre traits of the Gh and Gb panels in six environments.
**Table S3.** Correlation between six environments and heritability of nine fibre traits.
**Table S4.** Introgression test by three population statistics methods.
**Table S5.** Introgression events on a genome‐wide scale between the Gh and Gb groups.
**Table S6.** Significance test of nine fibre traits between the introgression and non‐introgression groups in the Gh and Gb panels.
**Table S7.** Genetic variation and diversity in the Gh panel.
**Table S8.** Genetic variation and diversity in the Gb panel.
**Table S9.** Selective sweeps in the Gh and Gb panels.
**Table S10.** Reported QTL overlapped with selective sweeps in the Gh panel.
**Table S11.** Linkage disequilibrium (LD) in the Gh and Gb panels.
**Table S12.** Genome‐wide association mapping in the Gh panel.
**Table S13.** Genome‐wide association mapping in the Gb panel.

## Data Availability

All relevant data can be found within the manuscript and its supporting materials.
